# *hbl-1* does not contribute to the misexpression of an adult cell fate marker in *daf-16* mutant dauer larvae

**DOI:** 10.17912/micropub.biology.001662

**Published:** 2025-06-24

**Authors:** Matthew J Wirick, Xantha Karp

**Affiliations:** 1 Biochemistry, Cell and Molecular Biology, Central Michigan University, Mount Pleasant, Michigan, United States; 2 Biology, Central Michigan University, Mount Pleasant, Michigan, United States

## Abstract

In *Caenorhabditis elegans* dauer larvae, the FOXO ortholog, *daf-16* , opposes the expression of the *col-19p::gfp* adult cell fate marker in the lateral hypodermis. *daf-16* acts in part via *lin-41 ,* a heterochronic gene that promotes larval seam cell fate during non-dauer development. Here, we show that a different heterochronic gene, *hbl-1* , does not function downstream of *daf-16* to regulate *col-19p::gfp* expression during dauer. A gain-of-function *hbl-1* allele did not suppress ectopic *col-19p::gfp* expression in *daf-16 (0)* dauer larvae, and HBL-1 protein was not detectable in control or *daf-16 (0)* dauer larvae.

**Figure 1. daf-16 does not regulate hbl-1 to control col-19p::gfp expression:**
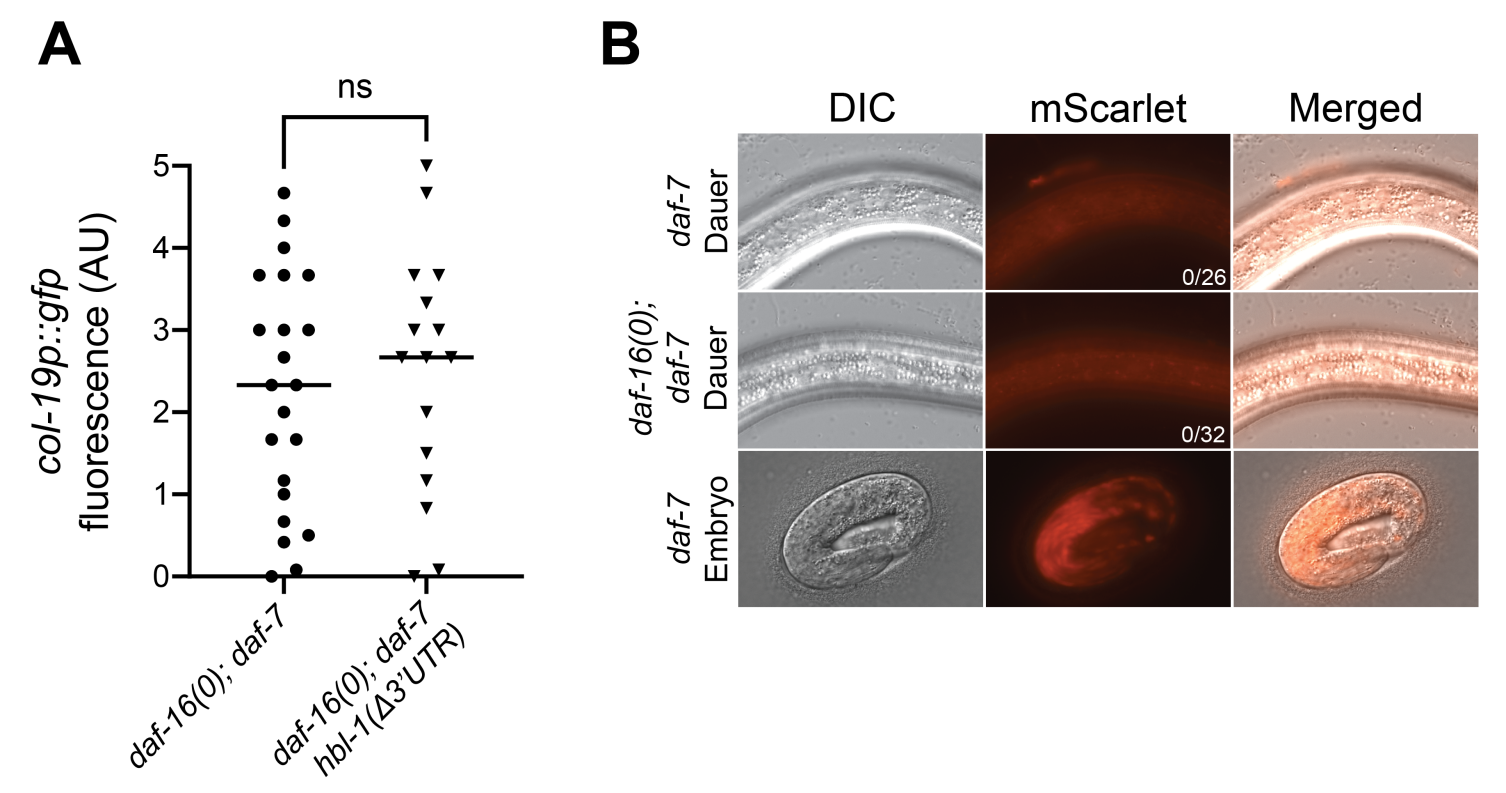
A) Deletion of the 3'UTR in *hbl-1* did not suppress the expression of *col-19p::gfp* in *daf-16 (0); daf-7* dauer larvae. Each data point represents the average fluorescence value for an individual dauer larva derived from three images taken the lateral hypodermis (see methods). p=0.6655, Mann-Whitney Test. (B) Representative DIC, mScarlet, and merged images of HBL-1 ::mScarlet expression. (Top & Middle) HBL-1 ::mScarlet expression was not detectable in *daf-7* or *daf-16 (0); daf-7* dauer larvae. (Bottom) HBL-1 ::mScarlet is visible in late *daf-7* embryos even at lower intensity settings than used for dauer larvae (see methods). Note that the embryos were not immobilized by levamisole and were moving inside the eggshell.

## Description

In *Caenorhabditis elegans ,* lateral hypodermal seam cells are multipotent progenitor cells that undergo a particular pattern and sequence of cell divisions at each larval stage until differentiating at adulthood (Sulston and Horvitz 1977). Adult seam cell fate includes the expression of adult-enriched collagens (Ambros and Horvitz 1984; Liu et al. 1995). In dauer larvae, seam cells remain quiescent and multipotent, despite a potentially lengthy period of developmental arrest (Liu and Ambros 1991). We have previously reported that the *C. elegans* ortholog of the Forkhead Box O (FOXO) transcription factor, DAF-16 , blocks adult cell fate during dauer (Wirick et al. 2021). Loss of *daf-16* ( *“ daf-16 (0)”* ) results in ectopic expression of the *col-19p::gfp* adult cell fate marker in dauer larvae. The regulation of *col-19p::gfp* by *daf-16* is at least partially mediated by positive regulation of *lin-41* , a heterochronic gene required for the temporal patterning of seam cell divisions in larvae during non-dauer development (Slack et al. 2000; Wirick et al. 2021). Though *lin-41* is required to block ectopic expression of *col-19p::gfp* during dauer, downregulation of *lin-41* via RNAi incompletely phenocopied the expression of *col-19p::gfp* in *daf-16 (RNAi)* dauer larvae. Additionally, a gain-of-function *lin-41* allele only partially suppressed the expression of *col-19p::gfp* in *daf-16 (RNAi)* dauer larvae (Wirick et al. 2021). Therefore, we hypothesized that *daf-16* may oppose *col-19p::gfp* expression through additional *lin-41* -independent mechanisms.

One candidate regulator of *col-19p::gfp* downstream of *daf-16* is the heterochronic gene *hbl-1 ,* which encodes the ortholog of the hunchback transcription factor (Fay et al. 1999; Abrahante et al. 2003; Lin et al. 2003). *hbl-1* and *lin-41* are both expressed early in larval development and promote stage-specific seam cell divisions. Later in development *,* *hbl-1* and *lin-41* are downregulated by *let-7 -* family microRNAs via their 3'UTRs (Reinhart et al. 2000; Abrahante et al. 2003; Lin et al. 2003; Abbott et al. 2005). *hbl-1 ,* like *lin-41 ,* can modulate the decision to enter dauer (Karp and Ambros 2011; Cale and Karp 2020). In addition, *hbl-1* has been implicated in the regulation of seam cell fate during the dauer life history as misexpression of *hbl-1* during or after dauer appears to cause heterochronic phenotypes (Karp and Ambros 2012).

To determine whether *hbl-1* plays a role in the expression of *col-19p::gfp* observed in *daf-16 (0)* dauer larvae, we first asked if a gain-of-function allele of *hbl-1* could suppress the *col-19p::gfp* expression phenotype. The gain-of-function allele of *lin-41* that suppresses this phenotype is a 3'UTR deletion that abrogates the microRNA-mediated downregulation of *lin-41* (Ecsedi et al. 2015; Wirick et al. 2021). We created a strain containing the *daf-16 ( mgDf50 )* null mutation and a large deletion in the *hbl-1* 3'UTR, *hbl-1 ( ma354 )* , that removes all predicted *let-7* complementary sites (Ilbay and Ambros 2019). We also included the *daf-7 ( e1372 )* allele to obtain populations of dauer larvae in dauer-defective *daf-16 (0)* mutants, as this mutation induces dauer formation in *daf-16 (0)* mutants at 24-25°C (Vowels and Thomas 1992; Wirick et al. 2021). We found *hbl-1 ( ma354 [∆3'UTR])* had no effect on the expression of *col-19p::gfp* in *daf-16 (0)* dauer larvae ( Fig. 1A ).

We next asked whether HBL-1 protein expression was altered in *daf-16 (0)* dauer larvae. Although our prior mRNA-seq data did not show a difference in *hbl-1* mRNA levels in *daf-16 (0); daf-7* vs. *daf-7* larvae (Wirick et al. 2021), levels or localization of HBL-1 protein could be altered in *daf-16 (0); daf-7* dauer larvae. Indeed, subcellular localization of HBL-1 is a known mode of regulation during continuous development (Ilbay and Ambros 2019). Furthermore, modENCODE data displayed on WormBase (WS296) show a DAF-16 binding peak upstream of *hbl-1* , suggesting that *daf-16* may regulate *hbl-1* expression in some contexts (Gerstein et al. 2010). However, the expression of an endogenously tagged *hbl-1* allele, HBL-1 ::mScarlet (Ilbay and Ambros 2019), was not detectable in either the *daf-7* or *daf-16 (0); daf-7* dauer larvae at exposure settings where HBL-1 ::mScarlet was detectable in embryos ( Fig. 1B ).

Taken together, we conclude that *daf-16* likely opposes the expression of *col-19p::gfp* during dauer independently of *hbl-1* . It will be interesting to explore other genes that may act in parallel to *lin-41* in future work.

## Methods

**Strains and maintenance**

*C. elegans* strains were grown and maintained at 15˚C or 20°C on Nematode growth media (NGM) plates seeded with *E. coli* strain OP50 as a food source (Brenner 1974).

**Dauer induction**

Dauer was induced at 24˚C using the *daf-7 ( e1372 )* allele which was present in all strains. For strains harboring the *hbl-1 (mScarlet)* endogenous tag, gravid adults were placed onto NGM plates seeded with OP50 and allowed to lay eggs for 3 hours at 24°C. Gravid adults were then removed from the plates. For *hbl-1 ( ma354 )* mutants, gravid adults were dissected to release fertilized eggs onto NGM plates seeded with OP50 . Populations of eggs on seeded NGM plates were then incubated at 24°C for 48-50 hours until dauer formation.

**Fluorescence microscopy**

Dauer larvae were mounted onto glass slides made with 2% agarose pads and paralyzed with 0.1M levamisole. A Zeiss AxioImager D2 compound light microscope with HXP 200 C fluorescent optics was used to image larvae. DIC and fluorescence images were captured with a AxioCam mRm Rev 3 camera and ZEN 3.2 software using a 63x objective. GFP images were captured with a Zeiss filter set 38HE and mScarlet images were captured with a Zeiss filter set 43 HE. The intensity of fluorescence can be altered on the HXP 200 C using a dial with 4 possible levels, where higher numbers indicate greater intensity. The expression of HBL-1 ::mScarlet in the representative embryo image was captured on the 2 ^nd^ intensity level of the HXP 200C exposed for 2000ms, while dauer larvae were imaged on the 3 ^rd^ intensity level and exposed for 2000ms.

**Phenotypic analysis**

The experimenter was blinded to genotype prior to imaging and analysis. *col-19p::gfp* expression was assessed using a semi-quantitative method (Wirick et al. 2021). Briefly, 3 images of *col-19p::gfp* were captured for each dauer larva along the lateral hypodermis. Each image was compared to existing representative images given a value 0.5-5 based off of the expression of *col-19p::gfp* , quantified by ImageJ. The average was taken from the three values for each worm and plotted. GraphPad Prism was used to create graphs and conduct the statistical analyses described in the figure legend. To determine the presence or absence of detectable HBL-1 ::mScarlet expression, three images were captured along the lateral hypodermis of dauer larvae and assessed for expression of HBL-1 ::mScarlet.

## Reagents

**Table:** 

Strain name	Genotype
XV36	*daf-16 ( mgDf50 ) I; daf-7 ( e1372 ) III; maIs105 [col-19p::gfp] V *
XV187	*daf-16 ( mgDf50 ) I; daf-7 ( e1372 ) III; maIs105 V; hbl-1 ( ma354 [∆3'UTR]) X *
XV232	*daf-16 ( mgDf50 ) I; daf-7 ( e1372 ) III; hbl-1 ( ma430 [ hbl-1 ::mScarlet-I]) X *
XV233	*daf-7 ( e1372 ) III; hbl-1 ( ma430 [ hbl-1 ::mScarlet-I]) X *
